# Prion protein facilitates synaptic vesicle release by enhancing release probability

**DOI:** 10.1093/hmg/ddu171

**Published:** 2014-04-09

**Authors:** Susan W. Robinson, Marie L. Nugent, David Dinsdale, Joern R. Steinert

**Affiliations:** 1 MRC Toxicology Unit, Hodgkin Building, Lancaster Road, Leicester LE1 9HN, UK; 2 Department of Genetics, University of Leicester, University Road, Leicester LE1 7RH, UK

## Abstract

The cellular prion protein (PrP^C^) has been implicated in several neurodegenerative diseases as a result of protein misfolding. In humans, prion disease occurs typically with a sporadic origin where uncharacterized mechanisms induce spontaneous PrP^C^ misfolding leading to neurotoxic PrP-scrapie formation (PrP^SC^). The consequences of misfolded PrP^C^ signalling are well characterized but little is known about the physiological roles of PrP^C^ and its involvement in disease. Here we investigated wild-type PrP^C^ signalling in synaptic function as well as the effects of a disease-relevant mutation within PrP^C^ (proline-to-leucine mutation at codon 101). Expression of wild-type PrP^C^ at the *Drosophila* neuromuscular junction leads to enhanced synaptic responses as detected in larger miniature synaptic currents which are caused by enlarged presynaptic vesicles. The expression of the mutated PrP^C^ leads to reduction of both parameters compared with wild-type PrP^C^. Wild-type PrP^C^ enhances synaptic release probability and quantal content but reduces the size of the ready-releasable vesicle pool. Partially, these changes are not detectable following expression of the mutant PrP^C^. A behavioural test revealed that expression of either protein caused an increase in locomotor activities consistent with enhanced synaptic release and stronger muscle contractions. Both proteins were sensitive to proteinase digestion. These data uncover new functions of wild-type PrP^C^ at the synapse with a disease-relevant mutation in PrP^C^ leading to diminished functional phenotypes. Thus, our data present essential new information possibly related to prion pathogenesis in which a functional synaptic role of PrP^C^ is compromised due to its advanced conversion into PrP^SC^ thereby creating a lack-of-function scenario.

## INTRODUCTION

The cellular prion protein (PrP^C^) is a cell membrane-anchored glycoprotein which plays an important role in a variety of neuronal processes including circadian rhythm, neuroprotection and neuroplasticity (1,2). Although the physiological role of PrP^C^ remains elusive, the conversion of PrP^C^ into the neurotoxic PrP^SC^ during prion disease and its signalling are well documented (2–4). As a consequence of protein misfolding, several mammalian species develop neurodegenerative conditions best known as scrapie in sheep, bovine spongiform encephalopathy in cattle or Creutzfeldt–Jacob disease (CJD) and Gerstmann–Sträussler–Scheinker Syndrome (GSS) in human. The unique feature of these conditions is that, in addition to sporadic and inherited forms, it can be transmitted by infectious agents according to the ‘protein only’ hypothesis. The early onset of disease may now be caused by either loss-of-function of PrP^C^ or gain-of-function of cytotoxic PrP^SC^, or both.

PrP^C^ is present in all mammalian cortico-cerebellar, deep nuclei neurons and neuromuscular junctions (NMJs) (5). Morphological studies suggest that PrP^C^ is preferentially located along axons and in presynaptic terminals (6) but postsynaptic localization and signalling has also been reported (7,8). Evidence accumulates that neuroprotective roles of PrP^C^ are essential (9,10) as loss-of-function in PrP^C^ knock-out (KO)/mutant models leads to neuronal dysfunction (11–13). Interestingly, KO animals for the gene encoding PrP^C^ exhibit phenotypes with impaired long-term potentiation (14–16), abnormal circadian rhythm (17) or effects on glutamatergic transmission (18,19) but also more severe characteristics such as Purkinje cell degeneration and demyelination of peripheral nerves leading to ataxia (11,20). As the exact cellular functions of PrP^C^ remain unknown, it is essential to characterize the physiological and neuroprotective roles of PrP^C^ in order to better understand the changes which occur during early onset prion disease.

Recently, several non-mammalian neurodegeneration models have been employed (21–23) and in particular, expression of PrP^C^ and PrP^SC^ in *Drosophila* or *Caenorhabditis elegans* allows investigations of prion function in host organisms that do not have a direct prion ortholog (24–29). PrP^C^ can convert into PrP^SC^ in adult *Drosophila* causing neurodegeneration and expression of a mutated PrP^C^ (PrP^P101L^) is sufficient to mimic neurodegenerative phenotypes in adult *Drosophila* (25,30). PrP^C^ can modulate synaptic transmission (31) including potentiation of acetylcholine release at the mouse NMJ (32), whereas PrP^C^-KO mice exhibit reduced inhibitory release (14). Research suggests that synaptic dysfunction precedes the cell death that occurs at later stages during prion pathogenesis (33,34) but studies have yet to define the exact physiological mechanisms of PrP^C^ in order to explain the underpinning synaptic loss and/or dysfunction before disease onset.

In the current study, presynaptic expression in *Drosophila* of mouse wild-type PrP^C^ (PrP^3F4^) and a mutated form of PrP^C^ [PrP^P101L^, which induces a GSS-like disease in mice and is related to a human GSS-associated mutation (P102L) (35)] was investigated to elucidate potential effects on synaptic release before manifestation of neurodegeneration thereby contributing to our understanding of PrP^C^ function. The data show that endogenous PrP^3F4^ facilitates synaptic release and this function is partially compromised following expression of PrP^P101L^ indicating a pivotal role of PrP^C^ (PrP^3F4^) signalling.

## RESULTS

### Expressed wild-type and mutated murine prion proteins are sensitive to proteinase digestion

**Figure 1.**
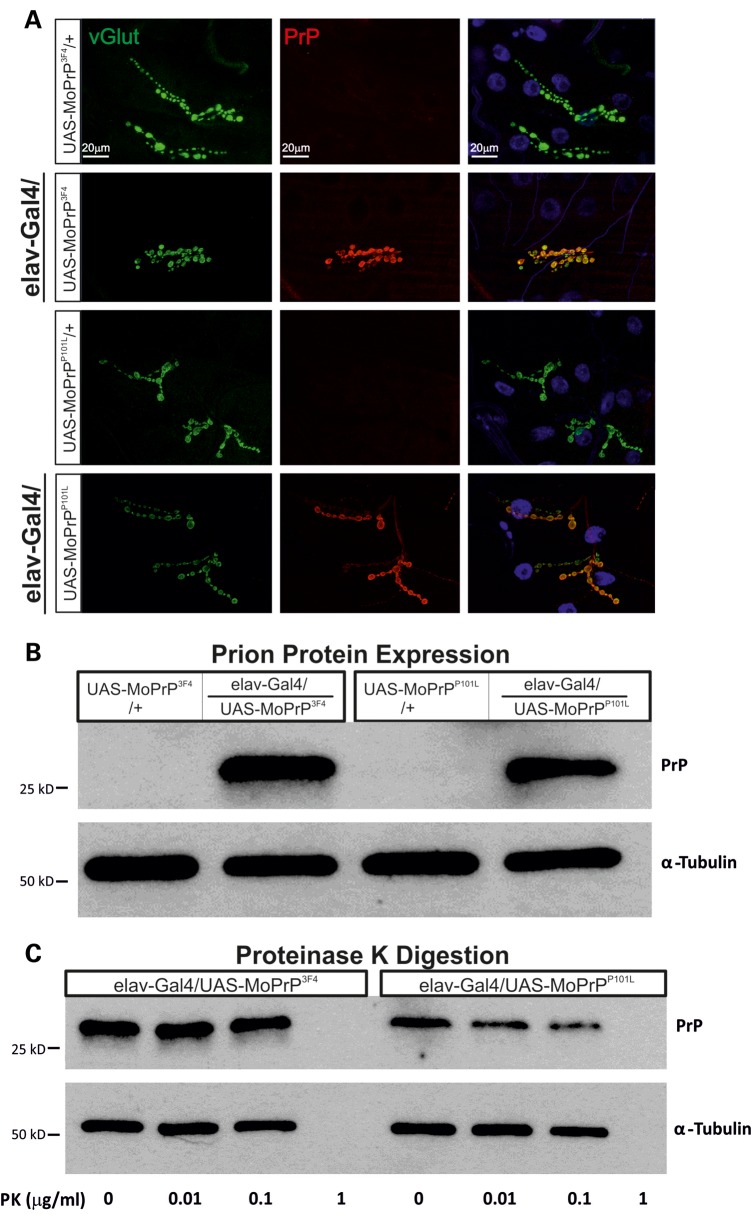
Prion protein expression does not result in PK resistance at the larval NMJ. (**A**) IHC staining of third instar NMJs show strong prion protein labelling in elav-Gal4/UAS-MoPrP^3F4^ and elav-Gal4/UAS-MoPrP^P101L^ larvae but no prion expression in respective UAS control NMJs. (**B**) Protein extracts of Tg-PrP lines (elav-Gal4/UAS-MoPrP^3F4^ and elav-Gal4/UAS-MoPrP^P101L^) with appropriate controls (UAS-MoPrP^3F4^/+ and UAS-MoPrP^P101L^/+) were probed for PrP (Ab: AH6 anti-PrP) and α-tubulin. Note, PrP^C^ can undergo glycosylation leading to multiple PrP bands (25,98) which are not detectable in larvae. *n* = 3 animals per lane. (**C**) Third instar larval extracts from Tg-PrP lines (elav-Gal4/UAS-MoPrP^3F4^ and elav-Gal4/UAS-MoPrP^P101L^) treated with a mild PK gradient (0–1 μg/ml) showed no PK resistance. PK completely digested prion protein (∼25 kDa bands) at relatively low concentrations (∼1 μg/ml) and so is α-tubulin being digested. *n* = 5 larval heads per lane.

Expression of wild-type murine PrP^C^ (PrP^3F4^) in *Drosophila* causes spongiform degeneration in adult fly brains (26) and importantly this degeneration is accelerated following expression of a mutated PrP^C^ (P101L) [PrP^P101L^], a mutation which is linked to the human prion disease GSS. In initial experiments we aimed to validate expression of either PrP^3F4^ or the mutated prion protein (PrP^P101L^) in transgenic *Drosophila* larvae by performing immunohistochemistry (IHC) which confirmed strong and specific expression of either protein within all boutons of the NMJ and lack of expression in UAS controls [Fig. 1A, co-stained for vesicular glutamate transporter (vGlut)]. Western blot analysis further confirmed expression of either prion protein (Fig. 1B). Assessing expression levels of both prion proteins revealed no differences between both lines (prion protein/α-tubulin ratio: PrP^3F4^: 1.2 ± 0.4 (*n* = 10), PrP^P101L^: 1.0 ± 0.2 (*n* = 7), *P* = 0.35, Student's *t*-test). As PrP^C^ expression induces a neurodegenerative phenotype in older adult flies (26,30) we next tested whether the expressed prion proteins were sensitive to proteinase digestion at these developmentally younger larval stages. In order to test this we employed the Proteinase K (PK)-digestion protocol. An increased resistance to PK digestion has been shown in older flies (30 days) expressing PrP^3F4^ where digestion occurs above ∼5–7 μg/ml (30,36) but it is unknown whether this resistance to PK digestion, an indicator of PrP^SC^ misfolding, is evident in third instar larvae. Any digestion occurring below ∼5–7 μg/ml could suggest a lack of misfolding and that the putative function of these proteins may not be affected (12). Incubation of larval preparations with various concentrations of PK (Fig. 1C, 0–1 μg/ml) did not indicate any formation of PK-resistant prion in either PrP^3F4^ or PrP^P101L^ transgenic lines as PrP^3F4^ and PrP^P101L^ digestion starts at ∼1 μg/ml (so is α-tubulin being digested by this concentration of PK, Fig. 1C). Thus, protein misfolding and aggregation is unlikely to occur at this developmental stage (12). It is established that synaptic loss in murine prion disease models precedes degeneration of the cell soma (34) but it remains to be investigated what the underlying mechanisms for this dysfunction are and whether pre- or postsynaptic prion protein signalling is involved. The current study aims to investigate prion protein-mediated effects on presynaptic release mechanisms to elucidate its physiological roles.

### Murine prion protein enlarges synaptic vesicles

**Figure 2.**
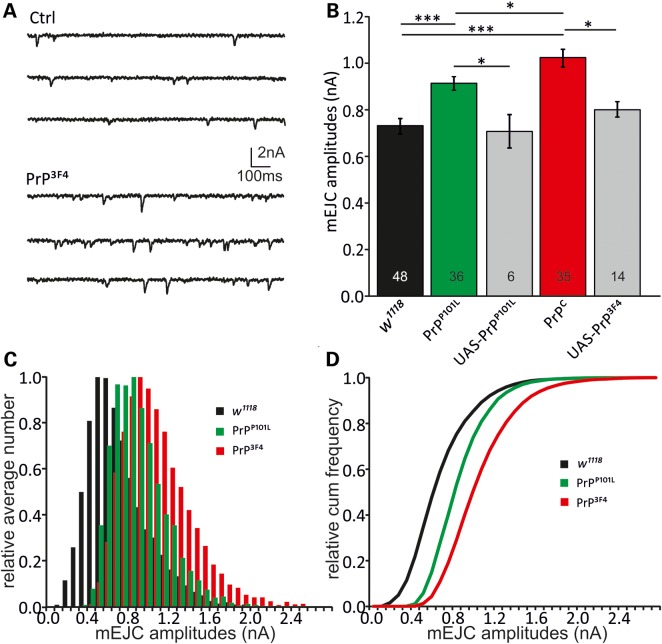
Prion causes enlarged miniature EJCs. TEVC recordings were performed at the NMJ to investigate the effects of neuronal PrP^3F4^ or PrP^P101L^ expression. (**A**) Sample traces showing mEJCs in control (Ctrl [*w^1118^*]) and PrP^3F4^ expressing larvae. (**B**) Mean mEJC amplitudes are increased following PrP^3F4^ and PrP^P101L^ expression with PrP^P101L^ showing a reduction relative to PrP^3F4^ (*n*—number of NMJs is indicated in bars. ***P < 0.001, *P < 0.05, *n* = 6–48 NMJs, [*n* = 19 animals for *w^1118^*, *n* = 15 animals for PrP^P101L^, *n* = 16 animals for PrP PrP^3F4^, *n* = 4–8 animals for UAS controls]). Data denote mean ± SEM, ANOVA with Tukey–Kramer *post hoc* test. (**C**) Normalized amplitude histograms for mEJCs show a right-shift of amplitudes following PrP^3F4^ expression. PrP^P101L^ expression left-shifted amplitudes relative to PrP^C^ but still caused an increase versus control (*w^1118^*). There is no evidence for multi-quantal release as distributions do not exhibit multiple peaks. (**D**) Relative cumulative amplitude histograms illustrate the right-shift in amplitudes for PrP^3F4^ expressing larvae. PrP^P101L^ expression induced a left-shift relative to PrP^3F4^ (K–S test, PrP^3F4^ versus *w^1118^*: *D* = 0.22, *P* = 0.024, PrP^P101L^ versus *w^1118^*: *D* = 0.30, *P* = 0.00023, PrP^3F4^ versus PrP^P101L^: *D* = 0.42, *P* < 0.00001). Distributions for UAS controls are not shown for simplicity.

To test the effects of PrP^3F4^ (pan-neuronal expression) on the synaptic physiology at *Drosophila* NMJs, Two-Electrode Voltage Clamp (TEVC) experiments were conducted to record miniature excitatory junctional currents (mEJCs). PrP^3F4^ expression induced a 38% increase in mean mEJC amplitudes compared with wild-type *w^1118^* (WT) controls [WT: 0.74 ± 0.03 nA (*n* = 48), PrP^3F4^: 1.02 ± 0.04 nA (*n* = 35), analysis of variance (ANOVA), *P* < 0.001, Fig. 2A and B]. The use of *w^1118^* as controls in addition to respective UAS controls was justified as all lines were backcrossed to *w^1118^* for at least six generations. This augmentation in mean mEJC amplitudes was also observed at NMJs expressing PrP^P101L^ (23%), although to a lesser degree then caused by the PrP^3F4^ expression (0.91 ± 0.04 nA (*n* = 36), *P* < 0.001 versus WT controls and *P* < 0.05 versus PrP^3F4^, ANOVA, *n*-number of NMJs indicated in bars). As additional controls, male larvae containing one copy of either UAS-PrP^3F4^ or UAS-P101L^D^ PrP^C^ [PrP^P101L^] were used (from now on referred to as UAS controls). Expression of either prion protein induced larger mEJCs relative to their respective UAS controls (*P* < 0.05 each, ANOVA). Each UAS control for PrP^3F4^ or PrP^P101L^ did not differ from WT (*w^1118^*) controls (UAS-PrP^3F4^: 0.84 ± 0.04 nA; UAS-PrP^P101L^: 0.71 ± 0.07 nA, *P* > 0.05, ANOVA). Comparison of mEJC amplitudes in WT controls of male and female larvae did not reveal any difference (male: 0.80 ± 0.07 nA (*n* = 8) versus female: 0.82 ± 0.07 nA (*n* = 5), *P* = 0.36, Student's *t*-test) consistent with no sex-dependence. The increase in mEJC amplitudes is due to a shift in the distribution of single quantal size as depicted in the normalized histograms and cumulative histograms for the three different genotypes. As shown in Figure 2C and D, normalized relative histograms for mEJC amplitudes and cumulative frequency histograms demonstrated an increased probability of larger amplitude events and hence a right shift in mEJC amplitude distributions in PrP^3F4^ but to a lesser degree in PrP^P101L^ expressing larvae. Using the more sensitive Kolmogorov–Smirnov test (K–S test) a difference between the distributions became evident (PrP^3F4^ versus Ctrl: *D* = 0.22, *P* = 0.024, PrP^P101L^ versus Ctrl: *D* = 0.30, *P* = 0.00023, PrP^3F4^ versus PrP^P101L:^ *D* = 0.42, *P* < 0.00001, K–S test) suggesting that functional properties between both prion proteins within the presynaptic terminal are different.

This PrP^3F4^-induced increase in mEJCs could either result from larger presynaptic vesicles or altered postsynaptic responses to a released vesicle. To test whether postsynaptic *Drosophila* glutamate receptor (*D*GluR) have functionally changed, kinetics of mEJC decays [indicative for *D*GluRIIA versus *D*GluRIIB contributions (37,38)] were compared. Mean decay tau (*τ*) values did not differ between genotypes nor did the *τ* distributions between tested conditions, indicating that *D*GluR composition had not changed (*τ*_Ctrl_: 6.1 ± 0.1 ms, *τ*_PrP_^3F4^: 6.5 ± 0.1 ms, *τ*_PrP_^P101L^: 6.2 ± 0.1 ms, *P* > 0.05, ANOVA). Further to this, the frequency (*f*) of mEJCs was not different between the three genotypes (*f*_Ctrl_ = 1.4 ± 0.1 s^−1^, *f*_PrP_^3F4^ = 1.7 ± 0.2 s^−1^, *f*_PrP_^P101L^ = 1.6 ± 0.2 s^−1^, *P* > 0.05, ANOVA) and comparable with published data (39,40) suggesting that the number of functional synapses responsible for Ca^2+^-independent release or basal release probability have not changed in response to prion protein expression. These data suggest that the prion protein-mediated changes are not due to postsynaptic alterations but most likely due to presynaptic changes in vesicular release.

**Figure 3.**
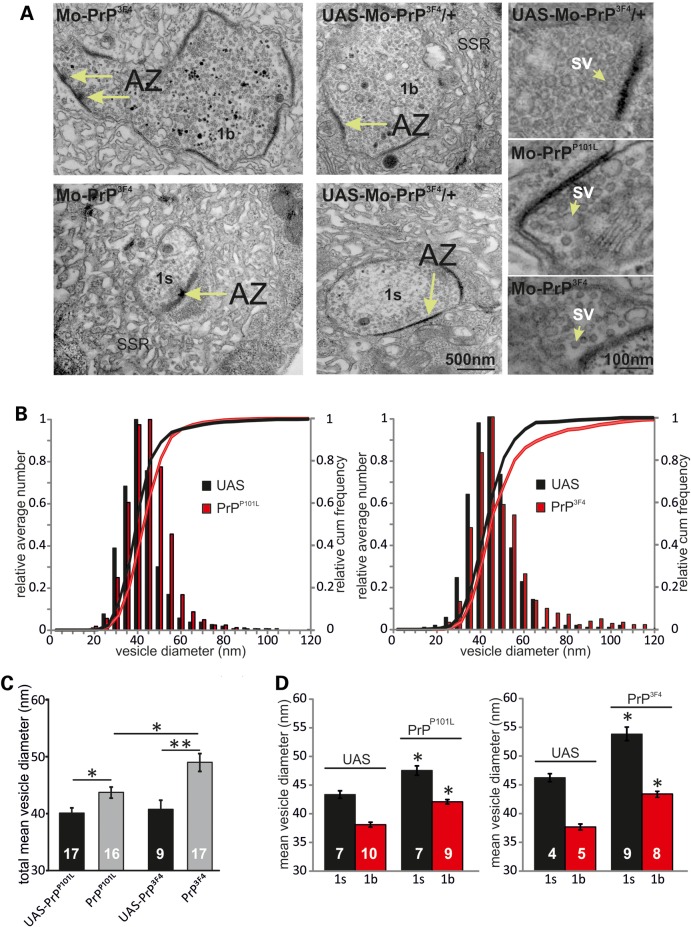
Prion causes enlarged presynaptic vesicles. (**A**) EM images for genotypes indicated. Images of synaptic boutons showing synaptic active zones (AZ—arrows with T-bars in left two images) and synaptic vesicles at higher magnification (SV—right three images for genotypes indicated). The boutons have been identified by the following features: 1b boutons are larger and possess more synapses, active zones and mitochondria compared with 1s. The enveloping sub-synaptic reticulum is more voluminous around type 1b boutons. (**B**) Relative average and cumulative histograms of total vesicle diameter counts including both 1s and 1b boutons (with similar percentage of both bouton types) in elav-Gal4/UAS-MoPrP^P101L^ (left) and elav-Gal4/UAS-MoPrP^3F4^ (right) larvae with their respective UAS controls. No difference in the mean number of AZ and T-bars between genotypes was detected. (**C**) Mean diameters of total vesicle counts including both 1s and 1b boutons (with similar percentage of both bouton types) for genotypes indicated showing increases of mean vesicle diameters following PrP^P101L^ and PrP^3F4^ expression. Note, PrP^3F4^ caused a greater increase relative to PrP^P101L^. (**D**) Left, mean vesicle diameters for 1s and 1b boutons from elav-Gal4/UAS-MoPrP^P101L^ and control UAS-MoPrP^P101L^/+ larvae. Right, mean vesicle diameters for 1s and 1b boutons from elav-Gal4/UAS-MoPrP^3F4^ and control UAS-MoPrP^3F4^/+ larvae. (PrP^3F4^: 46.2 ± 0.7 to 54.0 ± 1.1 nm [1 s]* and 37.7 ± 0.5 to 43.3 ± 0.6 nm [1b]*; PrP^P101L^: 43.7 ± 0.7 to 47.5 ± 0.8 nm [1s]* and 38.4 ± 0.4 to 42.1 ± 0.4 nm [1b]*; *P < 0.05, **P < 0.01, Student's *t*-test). Data denote mean ± SEM, *n*—number of boutons is indicated in bars [*n* = 3 animals for each genotype].

To further investigate synaptic vesicles at NMJ synapses we used electron microscopy (EM) to measure presynaptic vesicle sizes. As the *Drosophila* NMJ harbours two different kinds of boutons, namely 1s and 1b, both exhibiting different vesicular sizes, a subdivision of EM images into 1s and 1b boutons was made as shown before (41). The images in Figure 3A illustrate representative 1s and 1b boutons with release sites [long arrows: active zones (AZs)] and vesicles and higher magnification images for genotypes indicated. Bouton types were identified by size, number of synapses and AZs and size of the sub-synaptic reticulum (41) (Fig. 3A). Figure 3B shows average relative cumulative frequency histograms and histograms for vesicle diameters demonstrating an increased probability of larger diameters and hence can explain a right-shift in mEJC amplitude distributions in PrP^3F4^ and PrP^P101L^ expressing larvae relative to their respective controls (PrP^3F4^ versus UAS Ctrl: *D* = 0.131, *P* < 0.0001, PrP^P101L^ versus UAS Ctrl: *D* = 0.191, *P* < 0.0001, K–S test, *n* = 9–17 boutons). Furthermore, the difference between vesicle diameters from PrP^3F4^ and PrP^P101L^ boutons shows also significant differences (PrP^P101L^ versus PrP^3F4^: *D* = 0.137, *P* < 0.0001). Mean total vesicular sizes provide the best comparison to the mean mEJC amplitudes shown above (Fig. 2). Figure 3C illustrates the increase in mean total diameters in PrP^P101L^ (*n* = 16–17 boutons) and PrP^3F4^ larvae (*n* = 9–17 boutons) relative to their UAS controls but also the difference between PrP^P101L^ and PrP^3F4^ expressing larvae (*P < 0.05, **P < 0.01, Student's *t*-test). As these values are composed of 1s and 1b type boutons (equal number of each bouton type), we decided next to test whether prion protein effects could be observed at both bouton types. We subdivided the boutons according to previously published characteristics (number of synapses and AZs and size of the sub-synaptic reticulum) and vesicle diameter values for 1s and 1b boutons of ∼45 and ∼38 nm, respectively (41). Expression of either prion protein led to an increase in mean vesicle diameter in both types of boutons compared with their UAS controls (Fig. 3D, *P < 0.05, Student's *t*-test, *n* = 4–10 boutons). Interestingly, the number of AZs per bouton type did not differ between the genotypes (data not shown). The overall shift in the distribution of synaptic vesicle diameter in 1s and 1b boutons towards larger values suggests that PrP^3F4^/PrP^P101L^ is directly or indirectly involved in regulating vesicle size. Based on the changes in vesicular diameter we calculated the increase in volume which increased in PrP^3F4^ expressing larvae by 60 and 50% in 1s and 1b boutons, respectively, whereas PrP^P101L^ mutants showed an increase of 30 and 41% in 1s and 1b boutons, respectively. These increases are comparable with data from electrophysiological recordings although one has to consider an overestimation of measured mEJC amplitudes due to non-detectable smaller mEJCs (42). The data also imply that the mutated form of prion protein (PrP^P101L^) exhibits a diminished and altered function compared with PrP^3F4^ signalling. Together, this data suggest that PrP^3F4^ has an endogenous function in vesicle biogenesis and positively controls vesicle size and transmitter release at the *Drosophila* NMJ.

### Ca^2+^-dependent evoked release is enhanced by prion protein expression

**Figure 4.**
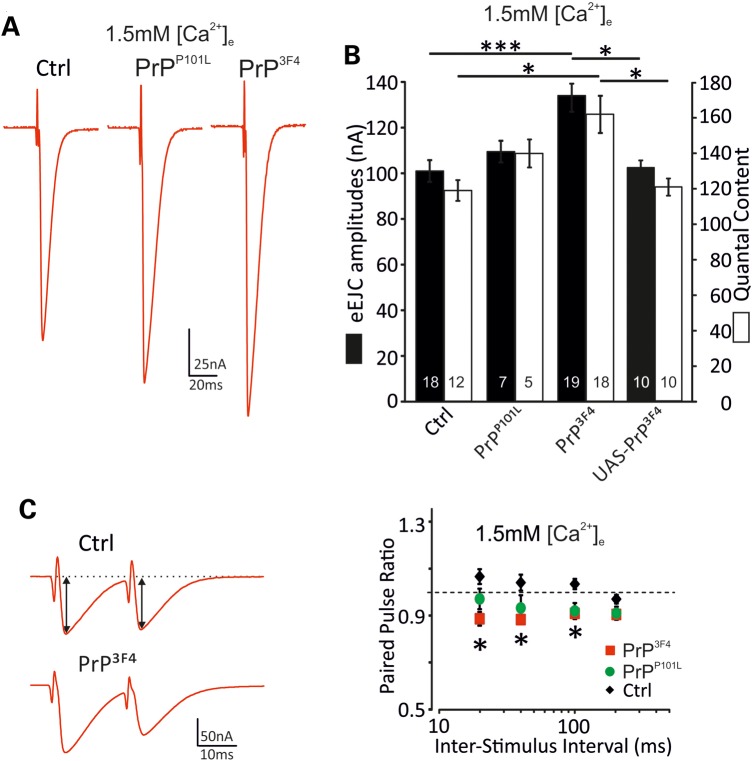
Evoked synaptic release and QC are enhanced by PrP^3F4^ expression. (**A**) representative eEJC recordings by stimulating the motor nerve in genotypes indicated at 1.5 mm [Ca^2+^]_e_. (**B**) Summary of mean eEJC amplitudes and QC for different genotypes at 1.5 mm [Ca^2+^]_e_, *P < 0.05, ***P < 0.001, ANOVA with Tukey–Kramer *post hoc* test, *n* = 5–19 NMJs (indicated in bars [*n* = 10 animals for controls, *n* = 5 animals for PrP^P101L^, *n* = 15 animals for PrP^3F4^, *n* = 6 animals for UAS-PrP^3F4^]). (**C**) Left, example traces for PPR for a Ctrl (*w^1118^*) and PrP^C^ NMJ at 20 ms inter-stimulus-intervals (ISI) at 1.5 mm [Ca^2+^]_e_. Black arrows indicate measurements of eEJC amplitudes. Right, summary of mean PPR versus ISI between 20 to 200 ms for three different genotypes. The dotted line indicates a PPR of 1 (20 ms: PPR_ctrl_: 1.07 ± 0.03, PPR_PrP_^3F4^: 0.89 ± 0.03*, PPR_PrP_^P101L^: 0.98 ± 0.04; 40 ms: PPR_ctrl_: 1.04 ± 0.03, PPR_PrP_^3F4^: 0.88 ± 0.02*, PPR_PrP_^P101L^: 0.94 ± 0.05; 100 ms: PPR_ctrl_: 1.04 ± 0.02, PPR_PrP_^3F4^: 0.91 ± 0.01*, PPR_PrP_^P101L^: 0.92 ± 0.03; 200 ms: PPR_ctrl_: 0.97 ± 0.02, PPR_PrP_^3F4^: 0.92 ± 0.01, PPR_PrP_^P101L^: 0.91 ± 0.01; ANOVA (*n* = 21 [Ctrl], *n* = 7 [PrP^P101L^], *n* = 13 [PrP^3F4^] NMJs [*n* = 10 animals for controls, *n* = 4 animals for PrP^P101L^, *n* = 7 animals for PrP^C^]), PrP^3F4^ versus Ctrl: *P < 0.05 at different ISI. ANOVA with Tukey–Kramer *post hoc* test. Data denote mean ± SEM.

The above data suggest a functional role of prion protein in synaptic function and vesicular release. To further investigate these effects on release mechanisms we measured evoked release at NMJs with presynaptic expression of PrP^3F4^ or PrP^P101L^. Evoked EJC (eEJC) amplitudes at low frequency stimulation (0.2 Hz) were enhanced following PrP^3F4^ (134 ± 5 nA*** (*n* = 19); *P* < 0.001, ANOVA) but not PrP^P101L^ expression (110 ± 9 nA (*n* = 7)) compared with *w^1118^* Ctrl (101 ± 5 nA (*n* = 18)) and UAS-PrP^3F4^ controls (103 ± 2 nA (*n* = 10)) suggesting a differential effect on synaptic transmission (Fig. 4A and B). eEJCs did not differ in their decay kinetics (data not shown) again confirming no alterations in postsynaptic receptor composition (37). Assuming that single quanta summate linearly in eEJCs and mEJCs and evoked responses arise from the same pool of vesicles at these low stimulus frequencies, quantal content [QC, the number of vesicles released per action potential (AP)] was estimated as the quotient of eEJC and mEJC amplitude per NMJ. The QC was strongly increased in PrP^3F4^ NMJs (*w^1118^* Ctrl: 119 ± 6 (*n* = 12); PrP^P101L^: 140 ± 8 (*n* = 5); PrP^3F4^: 162 ± 11* (*n* = 18); UAS-PrP^3F4^: 121 ± 5 (*n* = 10); *P* < 0.05, ANOVA, Fig. 4B). The enhanced QC could be attributable to an increase in either the number of release-ready vesicles or the release probability per vesicle.

To discriminate between these two options we initially analyzed paired pulse ratios (PPR) which critically depend on the initial release probability (*p*_vr_) (43) but also on the degree of facilitation (44) or on receptor saturation and desensitization (45,46). Receptor desensitization was excluded as eEJCs decay kinetics were identical between the first and last eEJC within a 1 s train (data not shown).

PPR provide a measure of *p*_vr_, in that the amplitude ratio of two closely spaced EJCs (EJC_2_/EJC_1_) increases as *p*_vr_ decreases (45). Here, the ratio of two eEJCs with inter-spike-intervals (ISI) varying from 20 to 200 ms was assessed at 1.5 mm [Ca^2+^]_e_. In all further experiments we used only *w^1118^* larvae as controls since responses in UAS-PrP^3F4^ controls and UAS-PrP^P101L^ controls did not differ from *w^1118^* (compare Figs 2 and 4B) and all strains were back-crossed for at least six generations to *w^1118^*. In agreement with previous data, control larvae showed weak paired-pulse facilitation at 100, 40 and 20 ms ISI (Fig. 4C) (42), whereas PrP^3F4^ expressing larvae showed significant depression at various ISI tested (ANOVA, *P* < 0.05, Fig. 4C) suggesting an increase in *p*_vr_ in PrP^3F4^ larvae and consistent with increases in QC following PrP^3F4^ expression (Fig. 4B). PrP^P101L^ again showed a non-significant tendency towards an increased *p*_vr_.

### Prion protein reduces synaptic vesicle pool size

**Figure 5.**
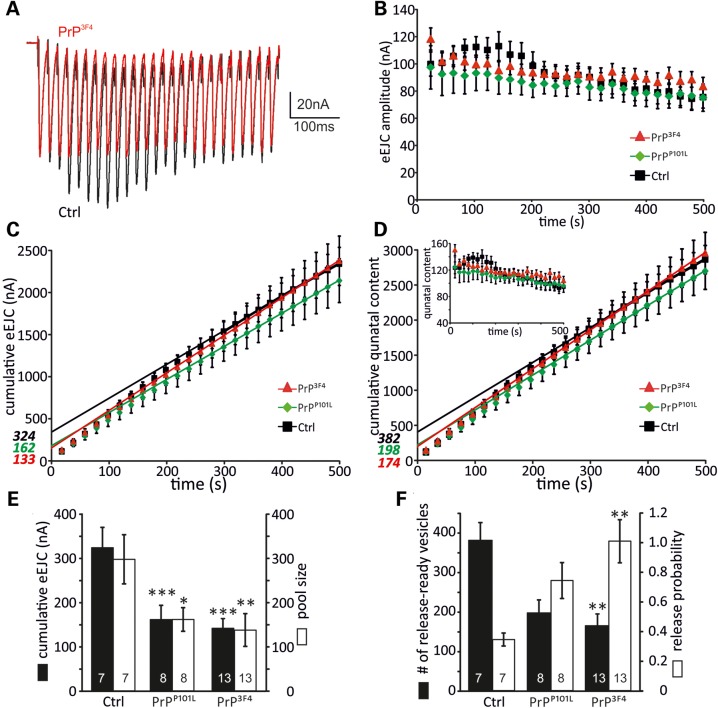
Number of readily releasable vesicles is reduced following PrP^3F4^ expression. (**A**) Example traces of 50 Hz trains (500 ms) from a Ctrl (black) and PrP^3F4^ expressing (red) larva at 1.5 mm [Ca^2+^]_e_. (**B**) Mean eEJC amplitudes from synaptic trains for the three different genotypes. (**C**) Cumulative eEJC amplitudes for the three genotypes with linear fits to the last 200 ms for each condition. Line fits to the cumulative eEJCs were back-extrapolated to time zero (indicated in italics, see Materials and Methods). (**D**) Average cumulative QCs with back-extrapolation of linear fits to the last 200 ms yielded estimates for the readily releasable pool (indicated in italics) for each genotype. Inset, mean QC during the 50 Hz stimulus train in the three genotypes indicated. (**E**) Mean values for cumulative eEJC amplitudes (black bars, ***P < 0.001) and vesicle pool sizes (open bars, *P < 0.05, **P < 0.01) are reduced compared with control. Pool size was determined by multiplying the cumulative eEJC amplitudes with the mean quantal size for this cell. (**F**) Mean number of ready-releasable vesicles (black bars, **P < 0.01) and the release probability for each genotype (open bars, **P < 0.001). The number of vesicles was estimated by dividing pool size by mean quantal size. The release probability was estimated by dividing eEJC_0_ by the pool size. ANOVA with Tukey–Kramer *post hoc* test (*n* = 7–13 NMJs as indicated in bars [*n* = 4 animals for controls, *n* = 5 animals for PrP^P101L^, *n* = 9 animals for PrP^3F4^]). Data denote mean ± SEM.

As changes in the observed PPR between control and PrP^3F4^ expressing synapses could be caused by alterations of two different parameters, namely: (i) the initial *p*_vr_ of the synapse but also (ii) the size of the ready-releasable pool (RRP) (43,47,48), it is important to estimate both of these synaptic parameters. A method to determine pool size was successfully applied at *Drosophila* NMJs by analyzing cumulative amplitudes within trains of higher frequency stimulation (42,49). Figure 5A shows 50 Hz trains (500 ms) of a control and PrP^3F4^ expressing NMJ. Control recordings show a strong initial potentiation followed by depletion which plateaued after ∼300 ms. In contrast, PrP^3F4^ expression did not allow potentiation within the train. Under these conditions, receptor desensitization was excluded as eEJCs decayed with identical time constants at the beginning and end of the train (data not sown). The average data of peak amplitudes revealed the difference between the genotypes as shown in Figure 5B. To check whether the observed differences are due to changes in *p*_vr_ and/or size of RRP we estimated these parameters (Fig. 5C–F). Stimulation at 50 Hz for 500 ms revealed a smaller cumulative eEJC amplitude (extrapolation to time point zero) of 162 ± 32 nA*** (*n* = 8) in PrP^P101L^ and 133 ± 20 nA*** (*n* = 13) in PrP^3F4^ expressing larvae compared with 324 ± 45 nA (*n* = 7) in control larvae (ANOVA, Fig. 5C and E) suggesting a strong reduction in pool size. The pool size was subsequently calculated by multiplying the cumulative amplitude by the mean quantal size of each muscle, confirming a strong reduction following PrP^3F4^ but also PrP^P101L^ expression (Fig. 5E). The number of release-ready vesicles was calculated by dividing the cumulative amplitude by the quantal size and was therefore reduced from control of 382 ± 44 (*n* = 7) to 174 ± 30** (*n* = 13) in PrP^3F4^ expressing larvae but not in PrP^P101L^ (198 ± 32 (*n* = 8), Fig. 5D and F; ANOVA). The possibility that recovery from depression was interfering with this data analysis could be excluded as it was shown previously that recovery occurs earliest at times >20 s (42).

Using this method, we further estimated the initial *p*_vr_ which can be calculated based on the cumulative amplitude analysis by dividing EJC_0_ of the train by the size of the RRP as described previously (50). The comparison between the three genotypes revealed that *p*_vr_ is increased following expression of PrP^3F4^ but not PrP^P101L^ in agreement with above data (Ctrl: 0.34 ± 0.04 (*n* = 7); PrP^P101L^: 0.74 ± 0.12 (*n* = 8); PrP^3F4^: 1.01 ± 0.14** (*n* = 13); Fig. 5F, ANOVA).

The above data consistently show that PrP^3F4^ enhanced *p*_vr_ and decreased the size of the RRP, whereas PrP^P101L^ only induced a decrease in the RRP size without affecting *p*_vr_.

**Figure 6.**
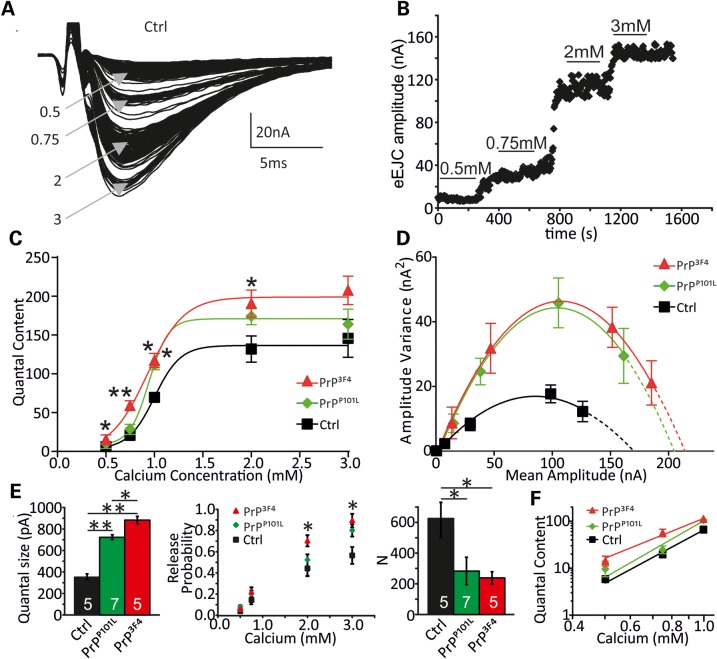
Prion expression induced changes in synaptic release parameters. (**A**) Group of eEJCs recorded from one NMJ at different indicated [Ca^2+^]_e_ in mm. (**B**) eEJC amplitudes of a PrP^3F4^ expressing NMJ elicited at 0.2 Hz at different [Ca^2+^]_e_. The lines indicate regions used for analysis. (**C**) Mean QC plotted versus different [Ca^2+^]_e_ for the genotypes indicated and fitted with a Hill function according to QC([Ca^2+^]) = QC_max_[1+(EC_50_/[Ca^2+^])^slope^]^−1^ which yielded QC_max_ = 136 ± 6, 171 ± 5 and 194 ± 15; EC_50_ = 1.0 ± 0.02 mm, 0.9 ± 0.01 mm and 0.9 ± 0.06 mm and Hill slope = 3.3 ± 0.5, 4.3 ± 0.3 and 2.4 ± 0.3 for Ctrl, PrP^P101L^ and PrP^C^ NMJs, respectively (Hill slope: PrP^3F4^ versus Ctrl: *P < 0.05, PrP^3F4^ versus PrP^P101L^: ***P < 0.001, data denote mean ± SD, ANOVA with Tukey–Kramer *post hoc* test, *n* = 8–11 NMJs [*n* = 5 animals for control, *n* = 6 animals for PrP^P101L^, *n* = 5 animals for PrP^3F4^]). Note, PrP^3F4^ causes a left-shifted [Ca^2+^]_e_—QC relationship but also and change in the slope of the fitted curves. (**D**) Parabolic fits to the variance–mean relationships for the three genotypes indicated. Note data are from a different subset of experiments as in C (data denote mean ± SEM). (**E**) Mean quantal parameters quantal size *q*, vesicular release probabilities (*p*_vr_) at different [Ca^2+^]_e_ and number of release-ready vesicles (*N*) estimated from fluctuation analysis in all three genotypes (*P < 0.05, **P < 0.01, ANOVA with Tukey–Kramer *post hoc* test, with *n*—number of NMJs indicated in bars [*n* = 4 animals for control, *n* = 4 animals for PrP^P101L^, *n* = 4 animals for PrP^3F4^], data denote mean ± SEM). (**F**) Double-logarithmic plot of the QC as a function of [Ca^2+^]_e_ for all three genotypes as indicated. Ca^2+^ dependency is decreased in PrP^3F4^ but not PrP^P101L^: controls (3.7 ± 0.4), PrP^C^ (2.7 ± 0.3*, *P* = 0.036), PrP^P101L^ (4.0 ± 0.9, *P* = 0.23), *n* = 20–27 NMJs [*n* = 12 animals for control, *n* = 11 animals for PrP^P101L^, *n* = 14 animals for PrP^C^]. Data denote mean ± SD, Student's *t*-test, ANOVA did not reveal any significance.

We next decided to apply a different and independent approach to estimate the synaptic parameters by which prion protein modulates synaptic release: fluctuation analysis (51–53) estimates the quantal parameters: *N*, *p*_vr_ and quantal size *q*. eEJCs were elicited at varying calcium concentration (0.5–3 mm, 0.2 Hz, Fig. 6A and B) and *N*, *p*_vr_ and *q* were estimated from parabolic fits to the variance–mean plots for each cell (Fig. 6D). By plotting the mean QC over various [Ca^2+^]_e_ (Fig. 6C) it became apparent that PrP^3F4^ expression led to enhanced release at 0.5–2 mm [Ca^2+^]_e_, whereas PrP^P101L^ expression only enhanced QC at 1 mm [Ca^2+^]_e_ (*P < 0.05, **P < 0.01, ANOVA). Data were fitted with a Hill equation yielding the Hill slope as a measure of Ca^2+^ co-operativity for prion protein expressing and control larvae. Interestingly, the Hill slope was reduced in PrP^3F4^ versus PrP^P101L^ and control NMJs from 3.3 ± 0.5 [Ctrl] and 4.3 ± 0.3 [PrP^P101L^] to 2.4 ± 0.3 [PrP^3F4^] (PrP^3F4^ versus Ctrl: *P < 0.05, PrP^3F4^ versus PrP^P101L^: ***P < 0.001, mean ± SD, ANOVA, Fig. 6) without affecting the half-maximal effective Ca^2+^ concentrations (EC_50_, ANOVA, *P* > 0.05, *n* = 8–11 NMJs) indicating that sensitivity to Ca^2+^ was not altered (54). A lower Hill slope has been associated with a tightening of the release site/Ca^2+^ channel complex (55,56) opening up the possibility for an interaction of PrP^3F4^ with Ca^2+^ signalling.

The fluctuation analysis further revealed, in agreement with data from mEJC measurements (Fig. 2), that *q* was enlarged following PrP^P101L^ (731 ± 8 pA, *n* = 7, *P* < 0.01) expression and even further increased in PrP^3F4^ (886 ± 24 pA, *n* = 5, *P* < 0.01 versus Ctrl and *P* < 0.05 versus PrP^P101L^) expressing larvae compared with controls (347 ± 27 pA, *n* = 5; Fig. 6E left, ANOVA). Furthermore, PrP^3F4^ had a stronger phenotype then PrP^P101L^ also in agreement with above data (Figs 2–4). The estimations further revealed an increase in *p*_vr_ at 2 and 3 mm [Ca^2+^]_e_ in PrP^3F4^ but not PrP^P101L^ expressing larvae although once again, PrP^P101L^ showed a tendency towards enhanced *p*_vr_ values (Fig. 6E middle) consistent with the enhanced QC and decreased PPR in PrP^3F4^ expressing larvae (Figs 4 and 5). On the other hand, the analysis showed that the number of release-ready vesicles decreased from 630 ± 104 (*n* = 5) in controls to 288 ± 91 (*n* = 7) in PrP^P101L^ and 246 ± 30 (*n* = 5) in PrP^3F4^ expressing larvae (Fig. 6E right, *P* < 0.05, ANOVA). Therefore, both, cumulative postsynaptic current and fluctuation analysis showed roughly a 50% reduction in those parameters following prion protein expression. The estimation of *N* from fluctuation analysis (∼600) in controls is in accordance with previously reported EM data showing a number of synapses onto muscle 6 of ∼500 (57). Together, these estimations are consistent with the data from above experiments where cumulative eEJC amplitude measurements (Fig. 5) or direct eEJC and mEJC measurements (Figs 2, 4 and 6) were conducted.

The enhanced QC seen in PrP^3F4^ expressing larvae can also be attributable to a change in Ca^2+^ dependence of release so we determined whether the elevated transmitter release is due to an altered Ca^2+^ co-operativity of release (58–60). The changes in the Hill slope shown above (Fig. 6C) indicate a reduction in Ca^2+^ co-operativity in PrP^3F4^ larvae but to corroborate this we analyzed the slopes of the linear regression lines to the double log QC–[Ca^2+^]_e_ relationship at lower non-saturating [Ca^2+^]_e_ (Fig. 6F). The data show that the Ca^2+^ co-operativity was reduced following PrP^3F4^ expression (*P* < 0.05, Student's *t*-test, Fig. 6) but not versus PrP^P101L^ (*P* > 0.05, Student's *t*-test, Fig. 6).

**Figure 7.**
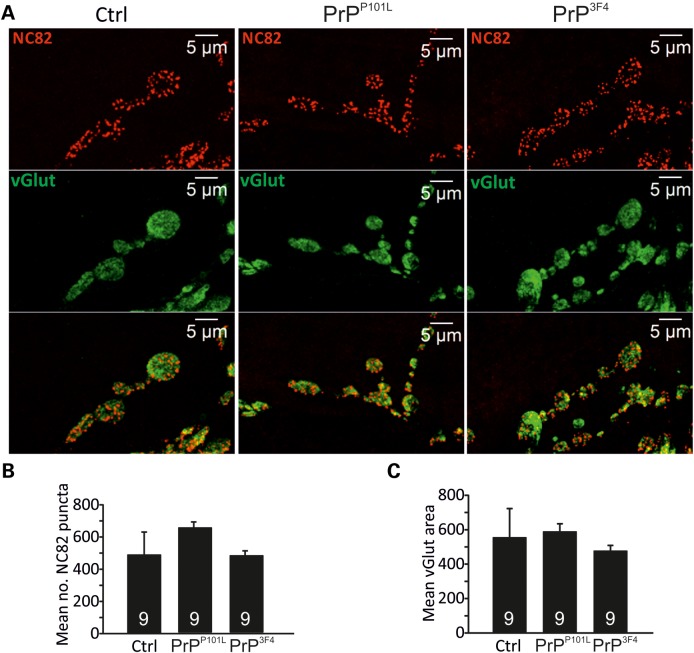
NMJ morphology is not affected by prion proteins. (**A**) Confocal images were taken from control, PrP^P101L^ and PrP^3F4^ expressing NMJs co-stained with anti-vGlut C-terminus and NC82 (anti-Brp) (Muscle 6). The number of NC82 puncta and vGlut area was calculated using Volocity software. (**B**) Mean data for counted NC82 puncta and vGlut area for the three genotypes indicated (*n* = 3 NMJs from three animals for each genotype, *P* > 0.05, data denote mean ± SEM. ANOVA with Tukey–Kramer *post hoc* test).

The detected reduction in the number of effective release sites as a consequence of prion protein expression could result from either functional or morphological alterations as developmental morphological changes at the NMJ are associated with various signalling pathways (61,62). To distinguish between these two possibilities we performed confocal imaging of NMJs and analyzed the number of NC82 puncta [Bruchpilot (Brp)—an essential component of the active zone cytomatrix T-bar] per NMJ and the NMJ size by calculating the area of vGlut staining. Figure 7 shows that both parameters are unchanged between the three genotypes (three NMJs from three larvae per genotype, *P* > 0.05, ANOVA) suggesting that prion protein expression does not induce any morphological changes and the reduction in release sites is due to functional rather than morphological changes.

### Prion protein mediates enhanced *Drosophila* larval locomotor activities

**Figure 8.**
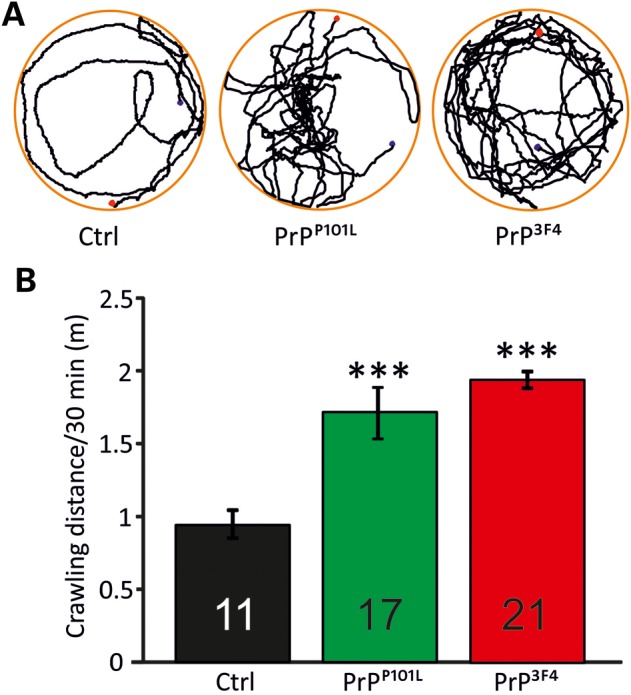
Prion proteins induce greater locomotor activities. Larval locomotor behaviour was recorded over a period of 30 min. Third instar larvae were put on a moist, food-free and temperature controlled (20°C) surface. Larval tracks were monitored (**A**, blue dot—starting point; red dot—end point of tracking) and data for each genotype were expressed as crawling distance (m) per 30 min (**B**) (*n* = 11 animals for control, *n* = 17 animals for PrP^P101L^, *n* = 21 animals for PrP^3F4^]). Data denote mean ± SEM (***P < 0.001, ANOVA with Tukey–Kramer *post hoc* test).

The outcome of the changes in synaptic transmission induced by prion protein expression would be a stronger depolarization of the postsynaptic muscle as long as the reduction in pool sizes does not become the limiting factor. On the behavioural level, a measure of low frequency muscle contraction would be the locomotor activity. The expectation would be that a stronger depolarisation leads to a stronger contraction and further locomotor distances. To test this behaviour, larvae were placed on a crawling device allowing on-line monitoring of individual larval activities (63). Locomotive impairment is a commonly used diagnostic for characterization of models for neurodegeneration where foraging behaviours are affected (64) and this test was employed to investigate locomotor activities of prion protein expressing larvae. Expression of either prion protein caused an increase in activity as detected in greater crawling distances per 30 min (*P* < 0.001, ANOVA, Fig. 8) relative to WT control larvae suggesting that the synaptic effects of prion protein expression also altered behavioural activities.

## DISCUSSION

Our data demonstrate a novel physiological function of prion protein at the synapse. Although many studies have reported neurodegenerative effects of misfolded prion protein, its physiological roles are much less understood. Initial data from mouse studies suggest that prion protein is required for synaptic transmission and mice lacking PrP^C^ show reduced excitatory and inhibitory synaptic currents. Various studies demonstrated a positive correlation between prion protein expression and synaptic transmission (14,18,32) suggesting that PrP^C^ is involved in neurotransmission (16,27), neurite outgrowth (65,66), circadian rhythm (17) and neuronal excitability (67). In the present study we have shown that PrP^3F4^ not only leads to larger vesicles but also increases *p*_vr_ with simultaneous reduction in the number of functional release sites equivalent to fewer ready-releasable vesicles. A mutated PrP^C^ (PrP^P101L^) exhibited diminished phenotypes illustrating a dysfunctional signalling.

As PrP^C^ expression causes an increase in vesicle diameter (gain-of-function), conversely, one would predict that deletion of prion protein signalling would induce the opposite effect. As the *Drosophila* genome does not contain an ortholog of mammalian PrP^C^, expression of the PrP^3F4^/PrP^P101L^ transgene induced the observed gain-of-function effect. In mammalian tissue, PrP^C^ is widely expressed and a loss-of-function would be observed in PrP^C^-KO mutants. In fact, PrP^C^-KO mice show reduced transmitter release at inhibitory synapses (14) and conversely, addition of PrP^C^ induces a potentiation of end-plate currents at mouse NMJs, most likely due to presynaptic mechanisms (32) supporting a physiological role of endogenous PrP^C^ in synaptic function. Recent evidence shows that PrP^C^ has physiological roles and hence loss-of-function effects due to conversion into PrP^SC^ or in PrP^C^-KO/mutant models were observed (11–13). PrP^C^ can interact with synapsin Ib, metabotropic glutamate receptors, Ca^2+^ channels or laminin, all of which represent potential binding partners for PrP^C^ (13,31,68,69). The role for PrP^C^–laminin interaction is consistent with scaffolding properties of PrP^C^ and its role in regulation of signal transduction (70) with altered PrP^C^ signalling leading to synaptic dysfunction.

The neurotoxic gain-of-function during prion disease is believed to be due to accumulation of PrP^SC^ but little evidence is available that loss of PrP^C^ plays a role during the pathogenesis. However, the possibility that prion diseases have loss-of-function components remains open (71,72) and a critical examination of this hypothesis depends on determining the elusive neuronal functions of PrP^C^. This current study took advantage of the *Drosophila* model by using the UAS/Gal4 bipartite expression system with pan-neuronal PrP^3F4^/PrP^P101L^ expression. The advantages of the *Drosophila* model to investigate neurodegenerative signalling pathways are well-recognized and several prion-related disease models in flies investigate ovine- (28) and mouse-prion protein (26,36) or GSS-mediated (25,30) neurodegeneration.

### Prion protein interaction with vesicle formation

Previous studies in mouse NMJs and hippocampal CA1 neurons suggested that PrP^C^ potentiates synaptic release (18,32) consistent with PrP^C^ expression at presynaptic terminals (73). However, pre- but also postsynaptic PrP^C^ signalling precludes a precise determination of its function. We have confirmed presynaptic actions of PrP^3F4^ showing an increase in the size of synaptic vesicles. An interaction between synaptic vesicles and PrP^C^ has been proposed at mammalian NMJs based on morphological studies (74) and our functional data support a role for PrP^C^ in vesicular homeostasis.

As PrP^C^ interacts with synapsin (31) and its internalization is mediated via clathrin-coated pits (75) in a dynamin-dependent process (76), one could speculate that PrP^C^ may play a role in endocytosis, vesicle replenishment and release. This interaction would offer a new functional explanation of how PrP^C^ modulates release processes at the synapse and how a consequent conversion of PrP^C^ into PrP^SC^ could induce synaptic dysfunction.

### Prion protein modulates release probability and vesicle pool size

Our findings that PrP^3F4^ expression leads to an increase in *p*_vr_ and decrease in the size of the functional readily releasable pool suggests further actions of PrP^C^ in regulating synaptic transmission. Changes in *p*_vr_ could be caused by alterations in Ca^2+^ channel density at release sites which occurs in mutants of Rab3 and Rab3-interacting molecule (RIM) (39,49,77), or by differential coupling of voltage-gated Ca^2+^ channels (VGCC) with synaptic vesicles (55,56). Furthermore, RIM-binding protein family members and the cytomatrix-associated protein Brp are essential in binding Ca^2+^ channels and loss of RIM or mutations in Brp lead to a loss of Ca^2+^ channels (clustering) within the AZ (78,79) illustrating an important connection between the architecture of AZs and Ca^2+^-mediated vesicle release.

In this context, a mutation in PrP^C^ leads to impaired membrane delivery of the α_2_δ-1 subunit of VGCC in cerebellar granule neurons (13) suggesting that PrP^C^ is required for intact synaptic Ca^2+^ signalling which may explain our results with respect to the increased *p*_vr_.

The number of release sites determined by fluctuation analysis (∼600) and postsynaptic current analysis (∼380) in our study is similar to the number of AZs reported previously using serial EM images (∼500) (57), current analysis (∼380) (42) or fluctuation analysis (∼500) (39). The fact that one AZ harbours not only one but two release-ready vesicles as shown at mouse NMJs (80) and following synaptic strengthening at *Drosophila* NMJs (42), allows for the variability of reported numbers of AZs. Importantly, in our study PrP^3F4^ expression caused a reduction in the number of release-ready vesicles with simultaneously enhanced *p*_vr_ as determined by independent methods demonstrating a synaptic role of PrP^C^.

Functional differences between single release sites have been reported at *Drosophila* NMJs indicating a specific and heterogeneous identity of each release site which determines its *p*_vr_ (81,82). This identity depends on clustering and the number and activation of VGCC as shown at the calyx of Held (83,84) or the structure and architecture of the cytomatrix (57,81). The organization of AZs is not static but undergoes plastic changes (85) and PrP^C^ could provide a potential candidate to interact with this structure. Our data point towards a model where prion protein expression results in fewer release sites with those remaining having greater *p*_vr_ possibly due to tighter coupling of Ca^2+^ channel/release site complexes by interacting with members of the AZ architecture. Our morphological studies indicate that PrP^3F4^ modulates the functionality rather than the number of AZs (Fig. 7).

### Is prion protein modulating calcium-mediated release?

Our findings that PrP^3F4^ expression alters the Ca^2+^ co-operativity as shown by a reduced Hill coefficient and slope of the log–log QC–[Ca^2+^]_e_ relationship (Fig. 6) supports the notion of an increased clustering of synaptic Ca^2+^ channels. At the mouse calyx of Held, synaptic strength increases with development (86,87) and this occurs in parallel with decreased Ca^2+^ co-operativity. The classical Ca^2+^ co-operativity usually refers to the co-operative interaction of 3–4 Ca^2+^ ions with the release machinery (58). Our data show similar co-operativity values in controls (∼3.3), whereas PrP^3F4^ exhibits a reduced value of ∼2.4. In contrast, the increase in co-operativity in PrP^P101L^ could reflect a crucial function of PrP^C^ in modulating Ca^2+^ signalling where this mutation shows a malfunctional phenotype. A reduced co-operativity and Hill slope (as seen with PrP^3F4^ expression) and increased synaptic strength are associated with tightening of VGCC to release sites in developmentally older compared with higher co-operativities and loosely coupled VGCC in younger synapses (55) suggesting that lower co-operativity, in a dynamic fashion, is associated with faster and more Ca^2+^-efficient synaptic responses (56). Physiologically, this lower co-operativity associated with higher *p*_vr_ may also reduce the effects of residual Ca^2+^ build-up and thus prevent depletion of synaptic vesicles allowing a reduction of release-ready vesicles whilst preserving the synaptic strength as shown in our data.

## CONCLUSION

In summary, based on the fact that PrP^C^ interacts with several proteins involved in synaptic release (31,76) and a range of ion channels, including VGCC (13), thereby modulating neuronal transmission, we investigated synaptic PrP^C^ signalling in more detail. Our data provide new evidence for a functional role of PrP^C^ in synaptic transmission where a modulation of vesicles and release properties by PrP^C^ increases synaptic strength. In relation to our findings, PrP^C^-mediated trafficking of VGCC subunits to the membrane which provides functional glutamatergic transmission (13), may play a crucial role in PrP^C^-mediated increases in synaptic release.

The molecular mechanisms of AZ organization are important determinants of synaptic function, in particular, proteins such as laminin β2 (scaffolding protein and PrP^C^ binding partner), Bassoon, CAST/Erc2/ELKS2α, Piccolo and RIMs (78,88) are essential components. In this context, it is worth highlighting the functions of Rab GTPases and RIM, which are crucially involved in synaptic transmission (22,77,78), and the facts that Rab3a activity is compromised in CJD (89) and Rab7a is a PrP^C^ interacting partner (90). This connection may present a signalling pathway by which PrP^C^ is required for intact Rab signalling, facilitating release, which will subsequently be diminished in prion disease resulting in synaptic dysfunction. Thus, the complex relationship between synaptic proteins and PrP^C^, with our data pointing towards an interaction of PrP^C^ with AZ function, suggests a novel physiological role. We propose a model, where prion protein signalling may lead to an optimization and increased efficiency of release, leading to higher Ca^2+^-dependent release probabilities with concurrent reduction in the number of enlarged release-ready vesicles. Further experiments to elucidate the exact interaction of PrP^C^ with the release machinery and synaptic ion channels, specifically molecules of the cytomatrix and Ca^2+^-mediated release, will allow a better understanding of the physiological roles of PrP^C^ signalling.

## MATERIALS AND METHODS

### *Drosophila* stocks

Flies were raised on standard maize media at 25°C. The *elav-Gal4* [c155] driver was obtained from the Bloomington Stock Center (Indiana, USA). The transgenic flies contain a UAS construct of either wild-type mouse prion protein on the third chromosome (*UAS-Mo-PrP^3F4^*) or mutant mouse prion protein (*UAS-Mo-PrP^3F4^* containing a proline-to-leucine substitution at residue 101) on the second chromosome (P101L^D^, referred to as P101L^D^ PrP^C^ [PrP^P101L^]) (25). Flies homozygous for the *UAS-PrP^3F4^* or *UAS-P101L^D^* constructs were crossed with flies homozygous for the elav-Gal4 driver to produce offspring expressing either PrP^3F4^ or mutant prion protein PrP^P101L^. The 3F4 epitope tag on Mo-PrP does not distort the normal topology or functions of PrP^C^ (91,92). The use of the UAS/Gal4 bipartite expression system to drive pan-neuronal expression excludes potential postsynaptic effects. All lines were backcrossed to *w^1118^* for at least six generations allowing the use of *w^1118^* as controls (Figs 5–8, unless otherwise stated) (39).

### Electrophysiology

TEVC recordings using sharp-electrodes were made from ventral longitudinal Muscle 6 in abdominal segments 2 and 3 of third instar larvae using pClamp 10, an Axoclamp 900A amplifier and Digidata 1440A (Molecular Devices, USA) in hemolymph-like solution 3 (HL-3) (60). Recording electrodes (10–30 MΩ) were filled with 3 m KCl. mEJCs were recorded in the presence of 0.5 μm tetrodotoxin (Tocris, UK). All synaptic responses were recorded from muscles with input resistances ≥4 MΩ and resting potentials more negative than −60 mV at 25°C as differences in recording temperature cause changes in glutamate receptor kinetics and amplitudes (93). Holding potentials were −60 mV. The extracellular HL-3 contained (in mm): 70 NaCl, 5 KCl, 20 MgCl_2_, 10 NaHCO_3_, 115 sucrose, 5 trehalose, 5 HEPES and 0.5–3 CaCl_2_ (as specified). Average single eEJC amplitudes (stimulus: 0.1 ms, 1–5 V) are based on the mean peak eEJC amplitude in response to ten presynaptic stimuli (recorded at 0.2 Hz). Nerve stimulation was performed with an isolated stimulator (DS2A, Digitimer). Paired-pulse experiments were performed by applying five repetitive stimuli (0.2 Hz) at different intervals (20, 40, 100 and 200 ms) for each cell at each inter-spike-interval. Materials were purchased from Sigma-Aldrich (UK) unless otherwise stated.

All data were digitized at 10 kHz and for miniature recordings, 200 s recordings we analyzed to obtain mean mEJC amplitudes, decay and frequency values. QC was estimated for each recording by calculating the ratio of eEJC amplitude/average mEJC amplitude followed by averaging recordings across all NMJs for a given genotype. mEJC and eEJC recordings were off-line low-pass filtered at 500 Hz and 1 kHz, respectively.

### Ca^2+^co-operativity

Ca^2+^ co-operativity was analyzed from synaptic current amplitudes recorded for each [Ca^2+^]_e_ from several muscles of different larvae. Co-operativity coefficients were derived by fitting linear regression lines to log-transformed individual data points for Ca^2+^ concentrations ≤1 mm and the slopes of the regression lines were statistically compared. Co-operativity coefficients estimated this way closely match coefficients derived by recording from several Ca^2+^ concentrations in single cells giving coefficients of ∼3–4 in wild-type larvae.

### Variance–mean analysis of eEJCs

Approximately 40 eEJCs were elicited at different [Ca^2+^]_e_, ranging from 0.5 to 3 mm to give mean eEJC amplitudes (I). The mean eEJC is given by *I* = *Np*_vr_*q* (52,94) with *N* being the number of independent release-ready vesicles, *p*_vr_ the vesicular release probability and *q* the quantal size at each given [Ca^2+^]_e_. The eEJC variance was calculated as previously described (52,95). The plots of the variance–mean were obtained for each cell and fitted with the parabolic function Var(*I*) = *I*^2^/*N* + *qI* that was constraint to pass through the origin. Upon fitting the parabola, *p*_vr_ and *q* were calculated using the equations: *q* = A/(1 + CV^2^) and *p*_vr_ = *I*(B/A)(1 + CV^2^) where CV^2^ is the coefficient of variation of the eEJC amplitudes at a given [Ca^2+^]_e_ concentration calculated as: CV^2^ = (eEJCs standard deviation/mean amplitude)^2^, A and B were obtained from the fitting parameters (53,96). Estimated values were not corrected for variability in mEJC amplitude distributions or latency fluctuations (51,97).

### Cumulative postsynaptic current analysis

The apparent size of the RRP was probed by the method of cumulative eEJC amplitudes (50), which was applied to the *Drosophila* NMJ previously (42). Muscles were clamped to −60 mV and eEJC amplitudes during a stimulus train (50 Hz, 500 ms) were calculated as the difference between peak and baseline before stimulus onset of a given eEJC. Receptor desensitization was not blocked as it did not affect eEJC amplitudes since a comparison of the decay of the first and the last eEJC within a train did not reveal any significant difference in decay kinetics. The number of release-ready vesicles was obtained by back-extrapolating a line fit to the linear phase of the cumulative eEJC plot (the last 200 ms of the train) to time zero. The number of release-ready vesicles was then obtained by dividing the cumulative eEJC amplitude at time zero by the mean mEJC amplitude recorded in the same cell. To calculate the QC in the train, we used mean mEJC amplitudes measured before the train.

### Larval locomotor assay

Age-matched third instar larvae (∼100–120 h) were selected, washed and placed onto a moist, food-free surface (with constant temperature of 20°C (63)). Crawling activities were imaged over 30 min using AnyMaze software v4.98 (Stoelting Co., USA) and data were analyzed off-line.

### Immunohistochemistry

Third instar larvae were dissected in ice-cold phosphate buffered saline (PBS) then fixed in 4% paraformaldehyde. After permeabilization with PBS-0.1% Triton (PBS-T) and blocking with PBS-T containing 0.2% bovine serum albumin and 2% normal goat serum, larval fillets were incubated at 4°C overnight in solutions of primary antibody. The following antibody dilutions were used: AH6 anti-PrP (TSE Reagent Resource Centre, Compton, UK) 1:1000 dilution; anti-vGlut C-terminus (kind gift from Hermann Aberle, University of Düsseldorf) 1:2000 dilution; NC82 (supernatant) anti-Brp (Bruchpilot; Developmental Studies Hybridoma Bank) 1:200 dilution and α-tubulin 1:4000 dilution. After 3 × 10 min washes in PBS-T, larvae were incubated with AlexaFluor 488 goat anti-rabbit and/or AlexaFluor 546 goat anti-mouse 1:1000 dilution for 90 min at room temperature. Larvae were mounted using Vectashield mounting medium (Vector Labs) and NMJ 6/7 (segments A2 and A3) images were acquired with a Zeiss laser-scanning confocal microscope (LSM 510, Carl Zeiss International). Image analysis was performed with ZEN (Carl Zeiss International) and Volocity software. Mean number of active zones per NMJ was calculated by dividing the number of NC82 (anti-Brp) puncta per NMJ by the total NMJ area detected by the vGlut antibody from maximum projections of *z*-stack images.

### PK digestion assay/immunoblotting

Five Third instar larvae heads were homogenized in 30 µl radioimmunoprecipitation assay buffer (150 mm NaCl, 10 mm Tris–HCl, 0.1% sodium dodecyl sulphate (SDS), 1% Triton X-100,1% sodium deoxycholate, 5 mm ethylenediaminetetraacetic acid) with Protease Cocktail Inhibitor (Promega) added. Homogenates were centrifuged at 4°C for 15 min at 16 000*g*. Ten micrograms of protein was incubated with the appropriate concentration of PK at 37°C for 30 min. The reaction was stopped by the addition of 2 mm phenylmethylsulfonyl fluoride, 4× Laemmli buffer was added and samples were boiled for 5 min before SDS-polyacrylamide gel electrophoresis and western blot analysis was performed. Prion protein aggregation (PrP^SC^) causes a lack of PK sensitivity resulting in digestion starting at ∼5–7 μg/ml relative to PrP^C^ digestion starting at ∼0.1 μg/ml.

### Electron microscopy

Third instar larvae were ‘filleted’ in phosphate-buffered saline at room temperature and then fixed in 2% (wt/vol) glutaraldehyde in 0.1 m sodium cacodylate buffer (pH 7.4) at 4°C overnight. They were post-fixed with 1% (wt/vol) osmium tetroxide/1% (wt/vol) potassium ferrocyanide for 1 h at room temperature and then stained *en bloc,* overnight, with 5% (wt/vol) aqueous uranyl acetate at room temperature, dehydrated and embedded in Taab epoxy resin (Taab Laboratories Equipment Ltd, Aldermaston, UK). Semi-thin sections, stained with toluidine blue, were used to identify areas containing synaptic regions (muscle 6/7 in regions A2/A3). Ultra-thin sections were cut from these areas, counterstained with lead citrate and examined in a JEOL 100-CXII electron microscope [JEOL (UK) Ltd, Welwyn Garden City, UK]. Images were recorded using a SIS Megaview III digital camera with iTEM software. SV measurements were made using ImageJ software. A total of ∼480–980 SVs were measured in 4–10 boutons from three animals per genotype.

### Statistics

Statistical analysis was performed with Prism 6 and InStat 3 (Graphpad, San Diego, USA). Statistical tests were carried out using an ANOVA test where applicable with *a posteriori* test or unpaired Student's *t*-test as indicated. Cumulative frequency distributions were compared using the Kolmogorov–Smirnov test (K–S test). Data are expressed as mean ± SEM or ± SD (as indicated) where *n* is the number of boutons (Fig. 3)/NMJs (Fig. 2 and 4–7)/larvae (Fig. 8) and significance is shown as *P < 0.05, **P < 0.01, ***P < 0.001.

## FUNDING

This work was supported by the Medical Research Council and BBSRC, UK. Funding to pay the Open Access publication charges for this article was provided by the Medical Research Council.
